# Integration of the Microbiome, Metabolome and Transcriptome Reveals *Escherichia coli* F17 Susceptibility of Sheep

**DOI:** 10.3390/ani13061050

**Published:** 2023-03-14

**Authors:** Weihao Chen, Xiaoyang Lv, Xiukai Cao, Zehu Yuan, Shanhe Wang, Tesfaye Getachew, Joram M. Mwacharo, Aynalem Haile, Kai Quan, Yutao Li, Wei Sun

**Affiliations:** 1 College of Animal Science and Technology, Yangzhou University, Yangzhou 225009, China; 2 Joint International Research Laboratory of Agriculture and Agri-Product Safety of Ministry of Education of China, Yangzhou University, Yangzhou 225009, China; 3 International Joint Research Laboratory in Universities of Jiangsu Province of China for Domestic Animal Germplasm Resources and Genetic Improvement, Yangzhou University, Yangzhou 225009, China; 4 International Centre for Agricultural Research in the Dry Areas, Addis Ababa 999047, Ethiopia; 5 College of Animal Science and Technology, Henan University of Animal Husbandry and Economics, Zhengzhou 450046, China; 6 CSIRO Agriculture and Food, 306 Carmody Rd, St Lucia, QLD 4067, Australia; 7 “Innovative China” “Belt and Road” International Agricultural Technology Innovation Institute for Evaluation, Protection, and Improvement on Sheep Genetic Resource, Yangzhou 225009, China

**Keywords:** *Escherichia coli* F17, lamb, metabolome, transcriptome, microbiome, omics integration, machine learning

## Abstract

**Simple Summary:**

*Escherichia coli* (*E. coli*) F17 is one of the major pathogenic bacteria responsible for diarrhea in farm animals; however, little is known about the biological mechanism underlying *E. coli* F17 infection. The aim of our study was to reveal the interplay between intestinal genes, metabolites and bacteria in *E. coli* F17 infected sheep. Our results confirm that the intestinal differ significantly in sheep with different *E. coli* F17 susceptibility, and integrated omics analyses reveal subsets of potential biomarkers for *E. coli* F17 infection (i.e., GlcADG 18:0-18:2, ethylmalonic acid and *FBLIM1*). Our results can help in the development of new insight for the treatment of farm animals infected by *E. coli* F17.

**Abstract:**

*Escherichia coli* (*E. coli*) F17 is one of the most common pathogens causing diarrhea in farm livestock. In the previous study, we accessed the transcriptomic and microbiomic profile of *E. coli* F17-antagonism (AN) and -sensitive (SE) lambs; however, the biological mechanism underlying *E. coli* F17 infection has not been fully elucidated. Therefore, the present study first analyzed the metabolite data obtained with UHPLC-MS/MS. A total of 1957 metabolites were profiled in the present study, and 11 differential metabolites were identified between *E. coli* F17 AN and SE lambs (i.e., FAHFAs and propionylcarnitine). Functional enrichment analyses showed that most of the identified metabolites were related to the lipid metabolism. Then, we presented a machine-learning approach (Random Forest) to integrate the microbiome, metabolome and transcriptome data, which identified subsets of potential biomarkers for *E. coli* F17 infection (i.e., GlcADG 18:0-18:2, ethylmalonic acid and *FBLIM1*); furthermore, the PCCs were calculated and the interaction network was constructed to gain insight into the crosstalk between the genes, metabolites and bacteria in *E. coli* F17 AN/SE lambs. By combing classic statistical approaches and a machine-learning approach, our results revealed subsets of metabolites, genes and bacteria that could be potentially developed as candidate biomarkers for *E. coli* F17 infection in lambs.

## 1. Introduction

*Escherichia coli* (*E. coli*) F17, a major subtype of enterotoxigenic *E. coli*, is one of the most common pathogens causing diarrhea in farm livestock, which causes huge economic loss and produces serious ecological concerns globally [1,2,3]. The *E. coli* F17 infection can disrupt intestinal tight junctions, increase permeability and resulting in severe vomiting, diarrhea and even death [4].

For the treatment of *E. coli* F17 infection, antibiotics are widely used in the farm industry; however, considering the potential of antimicrobial resistance [5], it is necessary to find alternative solutions, such as improving the susceptibility of animals to *E. coli* F17. To date, multiple approaches have been performed to understand the molecular signatures underlying pathogenic *E. coli* susceptibility of different species, such as RNA-seq [6], LC-MS [7] and GC-MS [8].

Moreover, various studies have shown that the integrated omics approach can identify the potential biomarkers that could be responsible for the *E. coli* infection. Fukuda et al. [9] integrated microbiome and metabolome analyses to elucidate the *E. coli* O157:H7 susceptibility of mice. In another study, integrated comparative genomics and immune-informatics approaches were applied to reveal the vaccine candidates against enterotoxigenic *E. coli* (ETEC) by Kusum et al. [10]. Recently, emerging evidence has indicated the promising power of machine-learning approaches in integrated omics studies for Alzheimer’s disease [11], cancer [12], diabetes [13], etc.

Within multiple machine-learning methods, Random Forest has shown high accuracy and a low overfitting risk for multi-omics datasets (features ≫ samples). Recently, several Random Forest analyses on multi-omics data have identified diverse biomarkers across multiple biological processes, such as colorectal cancer [14], quality traits of potato [15] and clear cell renal cell carcinoma [16]. Collectively, these mentioned studies highlighted the reliability of Random Forest approach in identifying potential biomarkers using different types of multi-omics dataset. However, only very few studies focused on the crosstalk between the different obtained datasets, especially on *E. coli* F17 infection.

In our previous study, lambs with different *E. coli* F17 susceptibility (*E. coli* F17-antagonism and -sensitive, AN and SE) were identified in a challenge experiment, and we analyzed the jejunal microbiota diversity [17] and transcriptomic profiles [18] using RNA-seq and 16s rRNA-seq approaches, respectively. The present study consists of two parts, first, the jejunal metabolite composition *E. coli* F17 AN and SE lambs were profiled using UHPLC-MS/MS approach. Then, we integrated the omics data to investigate the association between the microbiome, metabolome and transcriptome using a tree-based machine-learning approach: Random Forest. The aim of our study was to reveal the interplay between jejunal genes, metabolites and bacteria in *E. coli* F17 infection and could also help us understand the accuracy of this machine-learning method in integrated omics research.

## 2. Material and Methods

### 2.1. Sample Collection

Experimental lambs with different *E. coli* F17 (DN1401, fimbrial structural subunit: F17b, fimbrial adhesin subunit: Subfamily II adhesins, originally isolated from diarrheic calves) susceptibility were obtained from a challenge experiment—the details of the challenge experiment can be seen in our previous report [17].

In brief, 50 newborn Hu sheep lambs were randomly chosen and reared on lamb milk replacer free of probiotics and antimicrobial additives. The challenge experiment was conducted at 3 days after birth. The experimental lambs were divided into high-dose and low-dose challenge groups, which were orally gavaged with 50.0 and 1.0 mL of culture of *E. coli* F17 (1 × 10^9^ CFU/mL) for 4 days, respectively. Subsequently, the *E. coli* F17 susceptibility of experimental lambs was evaluated via stool consistency scoring, histopathological examination on intestinal tissue and bacteria plate counting of the intestinal contents.

Accordingly, six diarrhea lambs with severe intestinal pathology in the low-dose challenge group and six healthy lambs in high-dose challenge group were identified as *E. coli* F17-sensitive lambs (SE) and *E. coli* F17-antagonistic lambs (AN), respectively. Proximal jejunum tissue and jejunum contents were collected and stored in liquid nitrogen until use.

### 2.2. UHPLC-MS/MS Analysis and Data Processing

The collected jejunum contents were individually resuspended with prechilled 80% methanol. The resuspended tissues were incubated on ice for 5 min and then were centrifuged at 15,000× *g*, 4 °C for 20 min. Subsequently, the supernatant was diluted to a final concentration containing 53% methanol by LC-MS grade water and then were centrifuged at 15,000× *g* at 4 °C for 20 min.

Finally, the extracted jejunal metabolites were then accessed by the ultra-high-performance liquid chromatography–tandem mass spectrometry (UHPLC-MS/MS) analyses using a Vanquish UHPLC system (Thermo Fisher, Bremen, Germany) coupled with an Orbitrap Q Exactive™ HF-X mass spectrometer (Thermo Fisher, Bremen, Germany) [19]. Both positive polarity mode and negative polarity mode were operated to obtain the maximal coverage for jejunal metabolites.

The raw data generated by the UHPLC-MS/MS were processed using Compound Discoverer (Thermo Scientific, Waltham, MA, USA, version 3.1) to perform peak alignment, peak picking and quantitation for each metabolite [20]. Then, the peak intensities were normalized to the total spectral intensity. The normalized data were used to predict the molecular formula based on additive ions, molecular ion peaks and fragment ions. Subsequently, the peaks were matched with the mzCloud, mzVault and Masslist databases to obtain the accurate qualitative and relative quantitative results.

### 2.3. Identification of Differential Metabolites

These metabolites were annotated using the Kyoto Encyclopedia of Genes and Genomes (KEGG, https://www.genome.jp/kegg/pathway.html, accessed on 3 November 2021), Human Metabolome Database (HMDB, https://hmdb.ca/metabolites, accessed on 3 November 2021) and LIPID MAPS database (http://www.lipidmaps.org/, accessed on 3 November 2021). Principal components analysis (PCA) and partial least squares discriminant analysis (PLS-DA) were used to classify different samples with MetaboAnalystR R library ver 3.0 [21].

A *t*-test was used to identified subsets of differential metabolites, a metabolite was declared as differential metabolite if the difference in expression values between AN and SE lambs resulted in a Variable Importance in the Projection (VIP) > 1 and *p*-value < 0.05. The Pearson correlation coefficient (PCC) and *p*-value were also calculated to estimate the potential connection between differential metabolites, and the correlation was considered as statistically significant with a threshold of *p*-value < 0.05.

### 2.4. Acquisition of Microbiomic Dataset

The jejunal microbiota dataset used in this study was obtained from our previous research [17]; the raw data are available on: https://www.ncbi.nlm.nih.gov/, PRJNA827002, accessed on 10 October 2022.

In brief, total genome DNA was extracted from the jejunum contents of *E. coli* F17 SE/AN lambs, and the PCR amplifications of the 16S V3-V4 regions of the bacterial 16S rDNA gene were amplified using universal Primer 341F and Primer 806R. Sequencing libraries were prepared using TruSeq^®^ DNA PCR-Free Sample Preparation Kit (Illumina, San Diego, CA, USA) and sequenced on the Illumina NovaSeq platform after purification and quantification. After quality inspection, effective reads were assigned to the same operational taxonomic units (OTUs) by UPARSE software. The taxonomy of OTUs was aligned against SILVA reference database (SILVA SSU 138) based on the Mothur algorithm.

A total of 1115 OTUs were clustered and then assigned to 16 phyla, 31 classes, 79 orders, 127 families, 241 genuses and 163 species. The detailed information of and the results of differential abundance analysis can be found in Supplementary Table S1.

### 2.5. Acquisition of Transcriptomic Dataset

The mRNA expression dataset used in this study was obtained from our previous research [18]; the raw data are available on: https://www.ncbi.nlm.nih.gov/, PRJNA759095, accessed on 10 October 2022.

In brief, RNA was extracted from the jejunum tissue of *E. coli* F17 SE/AN lambs, and the mRNA libraries were prepared using a NEB Next^®^ Ultra™ RNA Library Prep Kit (Ipswich, MA, USA). All prepared libraries were sequenced on the Illumina HiSeq™ 2500 platform, and the raw data were obtained. After quality control, the clean reads were mapped to the *Ovis aries* reference genome (Oar_v4.0), and the mRNA candidates were distinguished using StringTie. A total of 20,601 mRNAs were screened. Differentially expressed (DE) mRNA were identified between AN and SE groups using edgeR, and 1465 DE mRNAs were identified between the AN and SE lambs. The detailed information can be found in Supplementary Table S2.

### 2.6. Integrated Analyses of the Multi-Omics Data Using Random Forest

Tree-based machine methods have shown promising results in identifying variable importance and interaction effects when applied to multi-omics data. In the present study, a tree-based machine-learning method (Random Forest) was conducted to investigate the interaction within multi-omics data; the detailed strategy for RF was described in our previous research [22].

In the present study, two integrated analyses were performed, namely Microbiome–Metabolome and Microbiome–Metabolome–Transcriptome. According to the omics data size, two key parameters (Ntree and mtry) were systematically examined. The out-of-bag (OOB) error rate was calculated to determine the minimum hyperparameter values required for subsequent analyses.

For interaction effect investigation, first, Random Forest was applied to select the subset of variables, and these variables were ranked by their values of variable important measures (VIM), the higher the “VIM” value, the more important the variable is in generate strong interactions with other selected variables [23]. Hence, the Pearson correlation coefficients (PCCs) between the top high “VIM” variables (the top 5% variables for Microbiome–Metabolome analysis and the top 2% variables for Microbiome–Metabolome–Transcriptome analysis) were calculated, and variable–target pairs with |PCC| > 0.8 and *p* < 0.05 were finally selected to establish the correlation network between different omics data.

The RF machine-learning analyses were conducted using the randomForest R package [24]. The networks were established using cytoscape software ver 3.9.1 [25].

## 3. Results

### 3.1. Profiles of Jejunal Metabolites

After filtering the internal standards and pseudo-positive peaks, a total of 1957 metabolites (1110 in positive polarity mode and 847 in negative polarity mode) were detected in the AN and SE samples. The details of the identified metabolites can be found in Supplementary Table S3.

**Figure 1:**
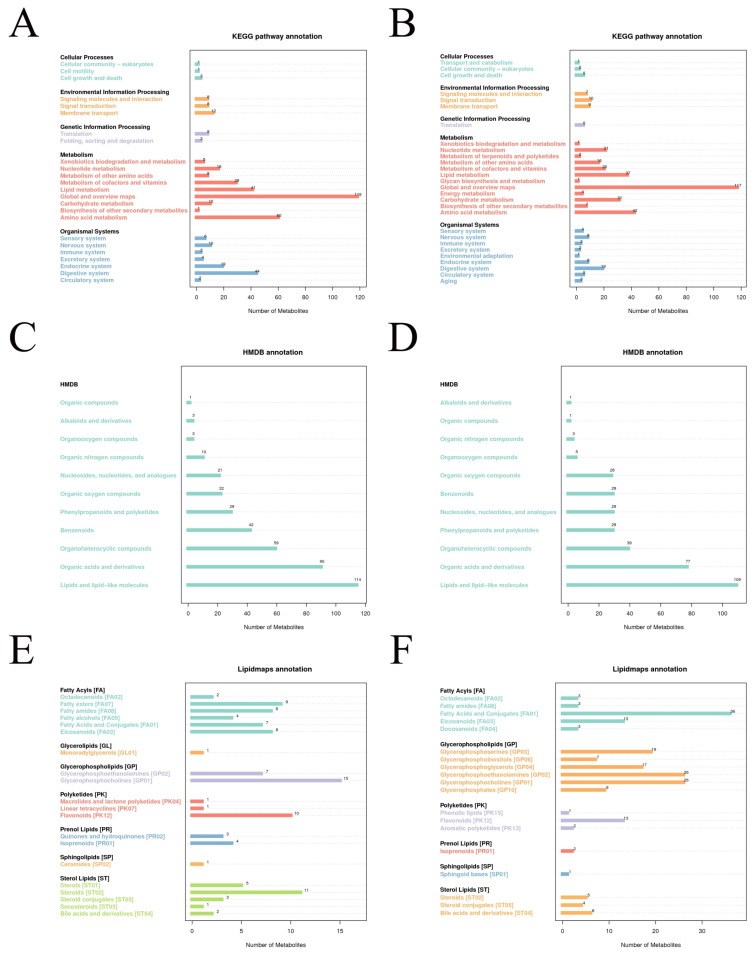
KEGG enrichment results of metabolites identified in positive polarity mode (**A**) and negative polarity mode (**B**). HMDB enrichment results of metabolites identified in positive polarity mode (**C**) and negative polarity mode (**D**). LIPID MAPS enrichment results of metabolites identified in positive polarity mode (**E**) and negative polarity mode (**F**).

To investigate the biological relevance of the identified metabolites, functional annotation was performed using the KEGG, HMDB and LIPID MAPS databases. Figure 1 present the annotation results of the identified metabolites, the top three enriched pathways from the KEGG enrichment analyses were Global and overview maps (Metabolism category), Digestive system (Organismal Systems category) and Lipid Metabolism (Metabolism category).

In HMDB annotation analyses, the top three enriched terms were Lipids and lipid-like molecules, Organic acids and derivatives and Organoheterocyclic compounds. In LIPID MAPS annotation analyses, the top three enriched terms were Fatty Acids and Conjugates, Glycerophosphocholines and Glycerophosphoethanolamines. The detailed annotation results of the identified metabolites can be found in Supplementary Table S4.

**Figure 2:**
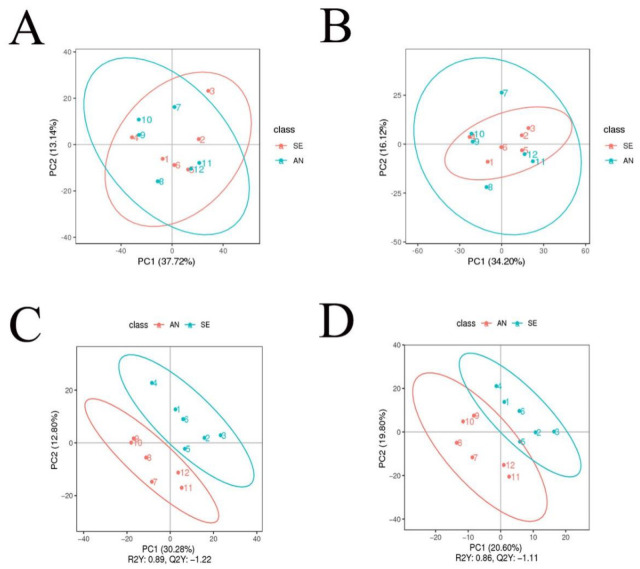
PCA scores plot of metabolites identified in positive polarity mode (**A**) and negative polarity mode (**B**). PLS-DA scores plot of metabolites identified in positive polarity mode (**C**) and negative polarity mode (**D**).

Subsequently, PCA and PLS-DA were utilized to classify different samples, the PCA scores plot (Figure 2A,B) showed that the difference between the metabolite profiles of AN and SE groups was not obvious; however, the PLS-DA scores plot (Figure 2C,D) showed that the AN and SE samples were divided into two groups. Collectively, our results indicated relatively different metabolite profiles of AN and SE groups.

### 3.2. Identification of Differential Metabolites

**Figure 3:**
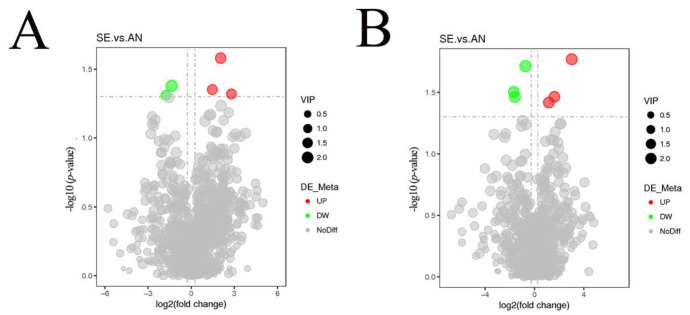
Volcano plot of differentially metabolites identified in positive polarity mode (**A**) and negative polarity mode (**B**).

Combining the *t*-test results and the VIP value calculated based on PLS-DA, five differential metabolites were identified between the AN and SE groups in positive polarity mode, within which three metabolites were upregulated, and two metabolites were downregulated. Six differential metabolites were identified between the AN and SE groups in negative polarity mode, within which three metabolites were upregulated, and three metabolites were downregulated (Figure 3). The detailed differential analyses results can be found in Supplementary Table S5.

**Figure 4:**
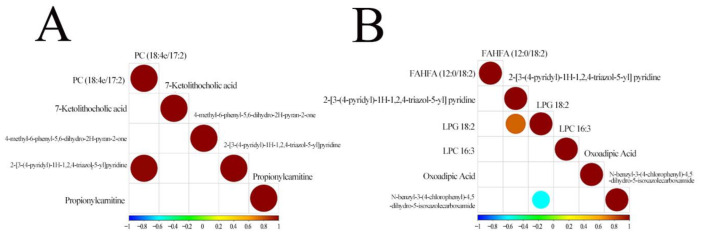
Correlation between the identified differentially metabolites in positive polarity mode (**A**) and negative polarity mode (**B**), where the deep color (red) and light color (blue) represent positive correlation and negative correlation, respectively.

The PCC and *p*-value were also calculated to estimate the potential correlation between the differential metabolites identified in positive polarity mode (Figure 4A) and negative polarity mode (Figure 4B), a strong positive correlation (PCC = 0.989) was observed between 2-[3-(4-pyridyl)-1H-1,2,4-triazol-5-yl] pyridine—PC (18:4e/17:2), and negative correlations (PCC = −0.596) were also observed between *N*-benzyl-3-(4-chlorophenyl)-4,5-dihydro-5-isoxazolecarboxamide and LPG 18:2.

### 3.3. Integrated Analysis of the Microbiome and Metabolome

For integrated analysis of microbiomic data and metabolomic data, all variables were first accessed by RF; the parameter training results and detailed RF results are provided in Supplementary Table S6.

The “VIM” values were calculated (supplementary table), and the top 5% variables (13 bacteria species and 93 metabolites) were selected for subsequent analysis. The top three variables with the highest “VIM” values were GlcADG 18:0-18:2 (15.85), 2-(3,4-dihydroxyphenyl)-7-hydroxy-3,4-dihydro-2H-1-benzopyran-4-one (13.41) and oxytetracycline (13.34).

**Figure 5:**
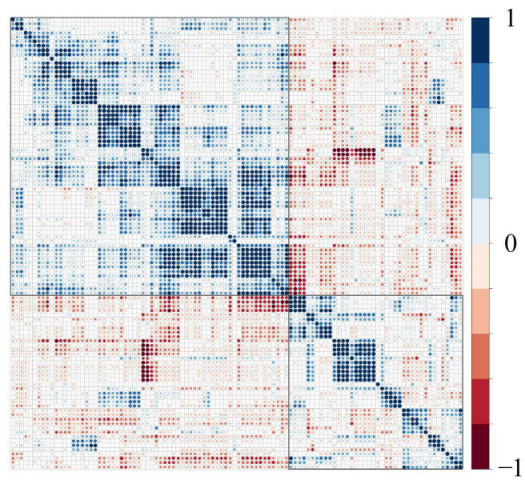
Correlation map of variables selected by RF for integrated analysis of the microbiome and metabolome, where the red color and blue color represent negative correlation and positive correlation, respectively.

**Figure 6:**
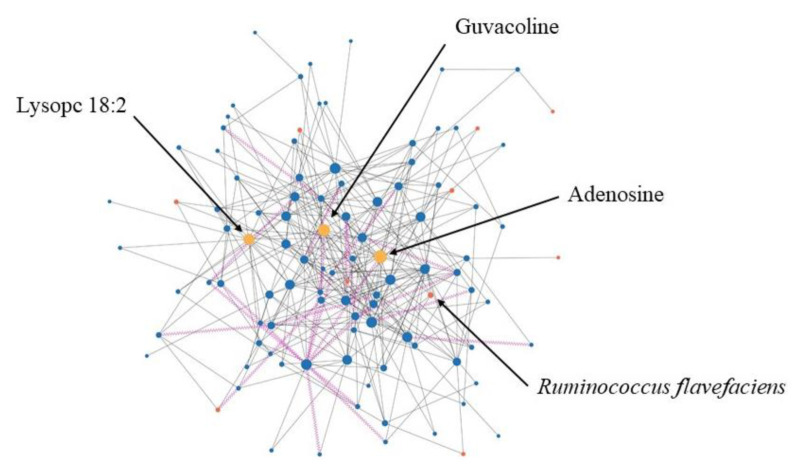
Interaction network of variables selected by RF for integrated analysis of the microbiome and metabolome, where solid lines (grey) and polylines (purple) represent positive correlation and negative correlation, respectively. The blue node and orange node represent metabolites and bacteria species, respectively. The top three nodes with the highest betweenness value are highlighted in yellow.

Subsequently, the PCC between the selected variables were calculated (Supplementary Table S7). The correlation map (Figure 5) indicates strong correlations between the selected variables, and 316 interaction pairs with |PCC| > 0.8 were finally obtained for interaction network construction (Figure 6).

For a better understanding of the interaction network, we calculated the edge betweenness centrality of each node in the network (Supplementary Table S8), the top three variables with the highest betweenness value were Adenosine (0.092), Lysopc 18:2 (0.085) and Guvacoline (0.083). The top bacteria species with the highest betweenness value was *Ruminococcus flavefaciens* (0.008), and the node with higher betweenness value indicated the node with stronger control power in the interaction network.

### 3.4. Integrated Analysis of the Microbiome, Metabolome and Transcriptome

The parameters training results for integrated analysis of the microbiome, metabolome and transcriptome are provided in Supplementary Table S6.

All variables were first accessed by RF, the “VIM” values were calculated (Supplementary Table S6), and the top 2% variables (307 genes, 145 metabolites and 2 bacteria species) were selected for subsequent analysis. The top three variables with the highest “VIM” value were Ethylmalonic acid (6.31), *FBLIM1* (5.89) and *RNF213* (5.73).

**Figure 7:**
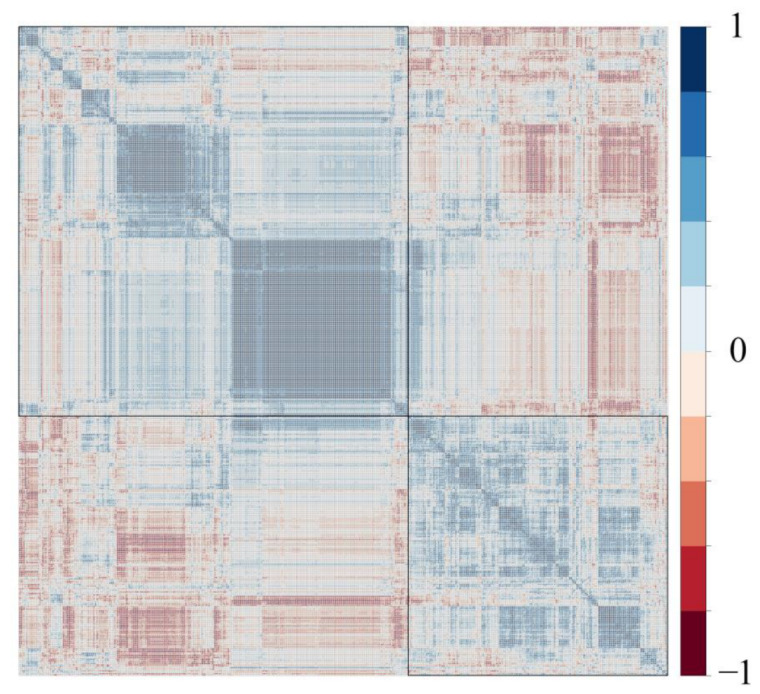
Correlation map of variables selected by RF for integrated analysis of the microbiome, metabolome and transcriptome, where the red color and blue color represent negative correlation and positive correlation, respectively.

**Figure 8:**
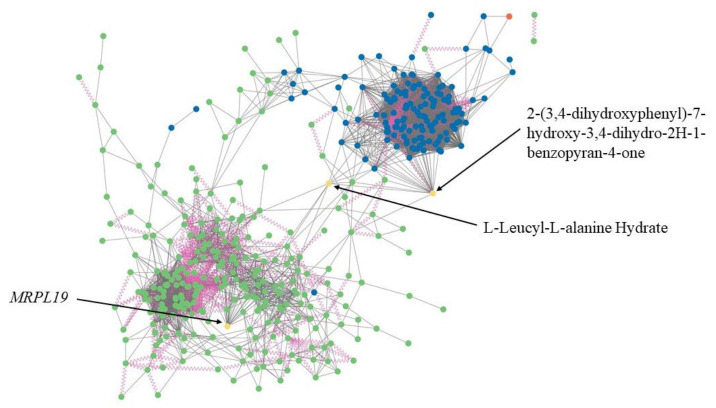
Interaction network of variables selected by RF for integrated analysis of the microbiome and metabolome, where solid lines (grey) and polylines (purple) represent positive correlation and negative correlation, respectively. The blue nodes, orange nodes and green nodes represent metabolites, bacteria species and genes, respectively. The top three nodes with the highest betweenness value are highlighted in yellow.

Subsequently, the PCCs between the selected variables were calculated (Supplementary Table S7). The correlation map (Figure 7) also indicated strong correlations between the selected variables, and 7779 interaction pairs with |PCC| > 0.8 were finally obtained for interaction network construction (Figure 8).

Regarding calculating the edge betweenness centrality of each node in the network (Supplementary Table S8), the top three variables with the highest betweenness value were L-leucyl-L-alanine hydrate (0.22), 2-(3,4-dihydroxyphenyl)-7-hydroxy-3,4-dihydro-2H-1-benzopyran-4-one (0.15) and *MRPL19* (0.13).

## 4. Discussion

Recent advances in ETEC reveal the metabolic [26], microbial [27] and genetic mechanisms [28] underlying ETEC infection in different species; however, little is known about the *E. coli* F17 susceptibility of animals. In the present study, the jejunal metabolite profiles of *E. coli* F17 AN and SE lambs were first investigated using UHPLC-MS/MS approach. In addition, we presented a multi-omics study using RF to gain insights into the complex interactions between the microbiome, metabolome and transcriptome in *E. coli* F17 challenged lambs.

### 4.1. Jejunal Metabolic Profile of E. coli F17 AN and SE Lambs

In the present study, a total of 1957 metabolites were profiled in AN and SE lambs, and a PCA plot and PLS-DA plot were constructed to classify different samples. The PLS-DA plot showed that a clear separation existed between the AN and SE lambs; however, clear group separation was not observed in the PCA plot. As a supervised dimension reduction method, PLS-DA can better yield group separation between similar groups compared with an unsupervised method (PCA) [29]. Considering the similar metabolic profile between *E. coli* F17 AN and SE lambs (all experimental lambs were challenged with *E. coli* F17), the different strategy underlying PCA and PLS-DA may be the reason for the different data grouping results.

Then, functional enrichment was performed on the identified metabolites. Pathways related to the lipid metabolism were found as mostly enriched. In the small intestine, lipids function to maintain the cellular integrity of the IECs [30]. Therefore, it is possible that *E. coli* F17 can affect intestinal metabolic homeostasis via regulating the lipid metabolism.

Subsequently, DE analysis was conducted to identify the differential metabolites, and 11 metabolites were identified differentially expressed in AN and SE lambs, within which, the most up-regulated metabolite was fatty acid esters of hydroxy fatty acids (FAHFA). FAHFAs are a kind of unique lipid messenger, which involves many immune-metabolic processes. In the gut, FAHFAs was previously proven to regulate GLP-1 (glucagon-like peptide-1) secretion and β-cell maturation [31]. Additionally, Rodriguez et al. reported that FAHFAs can inhibit apoptosis in colon carcinoma cells [32].

In the present study, the expression of FAHFA was remarkably higher in AN lambs than in SE lambs, which implied that the syntheses of FAHFA may also have similar inflammatory effects in sheep during *E. coli* F17 infection. The most down-regulated metabolite was propionylcarnitine. In humans, propionylcarnitine can serve as an immune marker to distinguish hepatocellular carcinoma from chronic hepatitis and cirrhosis [33].

In cattle, the upregulation of propionylcarnitine confirmed to confer good benchmarking for primary vaccine formulations [34]. Even though the biological effects of propionylcarnitine have so far not been fully characterized, the high expression of propionylcarnitine in SE lambs indicated that the assessment of propionylcarnitine may provide a feasible way to identify *E. coli* F17 AN individuals in lambs.

It is worth noting that only 11 metabolites were identified in the present study. Similar results were also obtained by He et al. [8] and Kim et al. [35], who found that only small subsets of metabolites were found to be differentially expressed between *E. coli* F18 challenged pigs and non-challenged pigs (serum, ileal mucosa and colon digest). These findings indicated that the relatively stable metabolic profile may be a global characteristic for ETEC infection, which presents the demand for a more efficient approach to discover metabolic biomarkers rather than classic statistical methods, such as the machine-learning approach.

### 4.2. Integrated Analysis of the Microbiome, Metabolome and Transcriptome

As a hallmark of ETEC, *E. coli* F17 infection can lead to the disrupted intestinal microbiota homeostasis, metabolic disorder and gene expression change [36]. In this context, we performed an integration of the microbiome, metabolome and transcriptome using RF, to investigate the crosstalk between genes, metabolites or bacteria species involved in the *E. coli* F17 infection.

Random Forest, as the leading class of machine-learning algorithms, has shown high accuracy and low overfitting risk in diverse biological analyses, especially for the high-dimensionality datasets, such as multi-omics data [37,38,39]. In RF analysis, a VIM value is generated for each variable accessed by RF, the higher the ‘VIM’ value is, the more important the variable is for generation a prediction in the decision trees [23]. Hence, selecting the variables with high VIM value can be an effective method for identifying important biomarkers in multi-omics study.

In the previous reported studies [39], 5% were usually set as the cutoff threshold for variables selecting in RF, considering the size of different omics datasets (163 bacterial species, 1957 metabolites and 20,601 genes), and 5% and 2% were set as the threshold for variables selecting in Microbiome–Metabolome analysis and Microbiome–Metabolome–Transcriptome analysis, respectively.

As mentioned above, the decision-tree-based strategy underlying RF indicated that certain interaction exists between the selected high-VIM variables and other variables [40]; hence, PCCs were calculated between selected variables. As expected, the correlation map of two multi-omics analyses showed that the selected variables are strong interacted with each other, which further prove the ability of RF in capturing the biological interaction between different omics datasets.

In the integrated analysis of the microbiome and metabolome, the top three variables with the highest “VIM” value were GlcADG 18:0-18:2, 2-(3,4-dihydroxyphenyl)-7-hydroxy-3,4-dihydro-2H-1-benzopyran-4-one and oxytetracycline. Oxytetracycline is a well-studied and widely used antimicrobial for treatment of various bacterial infections [41,42]; furthermore, Sarmiento et al. reported that oxytetracycline can decrease the adhesion of *E. coli* K88 to intestinal epithelial cells (IECs) [43].

Collectively, the present results showed that oxytetracycline may function similarly in lambs during *E. coli* F17 infection. Little is known about the roles of GlcADG 18:0-18:2 and 2-(3,4-dihydroxyphenyl)-7-hydroxy-3,4-dihydro-2H-1-benzopyran-4-one in the intestinal immune response during *E. coli* F17 infection; however, the high VIM value showed that these two metabolites may serve as potential biomarkers in *E. coli* F17 infection. In the interaction network analysis, the variable with the highest betweenness centrality was adenosine.

Adenosine is a key immunomodulator with complex biological roles in diverse immune responses [44,45]. Gross et al. reported that adenosine can protect mice against *E. coli*-induced acute lung injury [46]. However, Sun et al. reported that adenosine can also enhance the resistance of *E. coli* to acidic stress [47]. Despite the unexamined role of adenosine in *E. coli* F17 infection, the high betweenness centrality of adenosine implies that it may serve as a key regulator in the *E. coli* F17-induced diarrhea—of course, in-depth research needs to be performed to confirm our idea.

Regarding the integrated analysis of the microbiome, metabolome and transcriptome, the top three variables with the highest “VIM” value were ethylmalonic acid and *FBLIM1*. Interestingly, both of the top two variables have been proven to function important in intestinal homeostasis. Ethylmalonic acid is a metabolic organic acid and is highly correlated with intestinal permeability [48], while *FBLIM1* is a vital modulator in the epidermal growth factor receptor pathway [49], which can contribute to the proliferation and migration of IECs for preserving intestinal homeostasis [50]. The interaction analysis showed that the selected metabolites and genes were clearly separated into two groups, which were linked by L-leucyl-L-alanine hydrate, a commonly used substrate for fluorometric determination [51].

The gene with the highest betweenness centrality was *MRPL19*—directly linked to L-leucyl-L-alanine hydrate and 21 genes, which also has a high control power over the network. *MRPL19* is one of the house-keeping genes, with expression stability across different tissues [52]. At odds with their expression stability, in the present study, the expressions of L-leucyl-L-alanine hydrate and *MRPL19* in SE lambs were higher than that in AN lambs. Although the specific biological roles of L-leucyl-L-alanine hydrate and *MRPL19* remain incompletely understood, our results suggested that L-leucyl-L-alanine hydrate and *MRPL19* may function as vital regulators in intestinal immune response to *E. coli* F17 infection.

Regardless of the Microbiome–Metabolome analysis or Microbiome–Metabolome–Transcriptome analysis, only small subsets of bacterial species were picked in the analyses, the small size of the microbiome data (only 163 bacterial species) and similar intestinal microbiota between AN and SE lambs (all experimental lambs were challenged with *E. coli* F17), and this could be the reason for the results.

Additionally, the variables with the highest VIM value did not outperform other variables in interaction network analysis (betweenness centrality) as expected, one potential explanation for the results is that only the variables with the highest VIM were selected for the interaction network analysis, while massive interactions may exist between the top variables and the other unselected variables. Another explanation is that the top variables with the highest betweenness centrality might be considered as leaders of connections to the *E. coli* F17 susceptibility, while the top variables with the highest VIM were directly involved in that.

## 5. Conclusions

In summary, our study provides the metabolomic profile of *E. coli* F17 AN and SE lambs. A total of 1957 metabolites were profiled in the present study, and 11 differential metabolites were identified. Then, the Random Forest method was conducted to integrate the microbiome, metabolome and transcriptome data of *E. coli* F17 AN and SE lambs, and several potential biomarkers for *E. coli* F17 infection (i.e., GlcADG 18:0-18:2, ethylmalonic acid and *FBLIM1*) were identified. Moreover, the interaction network was constructed to gain insight into the crosstalk between the genes, metabolites and bacteria in *E. coli* F17 AN/SE lambs. The results can help us to understand the underlying mechanisms of *E. coli* F17 susceptibility at the integrated omics level.

## Data Availability

The metabolomic datasets presented in this study can be found in online repositories. The names of the repository/repositories and accession number(s) can be found below: https://www.ebi.ac.uk/metabolights/MTBLS7061, accessed on 2 January 2023.

## Supplementary Materials

The following supporting information can be downloaded at: https://www.mdpi.com/article/10.3390/ani13061050/s1, Table S1: Microbiomic data of AN and SE samples. Table S2: Transcriptomic data of AN and SE samples. Table S3: Detailed identified metabolites of AN and SE samples. Table S4: Annotation results of the identified metabolites. Table S5: Differential analyses results of the identified metabolites. Table S6: Parameters training and detailed results of Random Forest. Table S7: PCCs between the variables selected by Random Forest. Table S8: Edge betweenness centrality of each node in the interaction network.
